# Let‐7e as Potential Diagnostic Biomarkers in Early Detection of Peri‐Implant Diseases: A Cross‐Sectional Study

**DOI:** 10.1002/cre2.70044

**Published:** 2025-05-05

**Authors:** Nurul Latifah Zainal Abidin, Norjehan Latib, Fouad Hussain Al‐Bayaty, Mohd Faizal Hafez Hidayat

**Affiliations:** ^1^ Centre of Periodontology Studies University Technology MARA Sungai Buloh Campus, Jalan Hospital Sungai Buloh Malaysia; ^2^ Periodontic Unit Kota Damansara Dental Clinic Petaling Jaya Malaysia; ^3^ Department of Postgraduate Study Malaysia MAHSA University Jenjarom Malaysia

**Keywords:** ieri‐implantitis, let‐7e, miRNA biomarkers, peri‐implant crevicular fluid, peri‐implant diseases, peri‐implant mucositis, real‐time polymerase chain reaction

## Abstract

**Objectives:**

Peri‐implant diseases (PID), specifically peri‐implant mucositis and peri‐implantitis, significantly jeopardize dental implant success. Traditional diagnostic methods, relying on periodontal probing and radiographs, have limitations in precisely diagnosing these conditions. Recent studies suggest that microRNA (miRNA) profiling might be a breakthrough in the early detection of PID. This study aimed to evaluate the expression levels of specific miRNAs—miR‐98, miR‐145, miR‐146a, and let‐7e—in individuals with peri‐implant mucositis and peri‐implantitis compared to a healthy control.

**Methods:**

From January 2022 to October 2023, 19 participants with 38 dental implants were recruited according to established criteria. After clinical and radiographic evaluations, dental implants were grouped as peri‐implant health (Group 1), peri‐implant mucositis (Group 2), and peri‐implantitis (Group 3). Subsequently, peri‐implant crevicular fluid (PICF) samples were collected and analyzed through real‐time quantitative polymerase chain reaction (qPCR).

**Results:**

Based on the findings, only let‐7e biomarkers were consistently detected across all samples, with their expression increased eightfold in peri‐implant mucositis and 94‐fold in peri‐implantitis cases. Kruskal–Wallis's test showed a statistically significant difference in relative gene expression of let‐7e between the different groups, *H*(2) = 25.825, *p* ≤ 0.001.

**Conclusions:**

Our study indicates that miRNA biomarkers, particularly let‐7e, may play a significant role in the development and progression of PID, highlighting the need for further in‐depth investigation, with PICF offering a practical, noninvasive sampling method.

## 1 Introduction

Osseointegrated dental implants have become the preferred treatment modalities to replace missing teeth due to their high survival rates and positive patient outcomes, with studies reporting survival rates up to 96.4% over periods extending to 10 years (Howe et al. 2019). Despite the relatively high long‐term survival rate, biological complications, namely, peri‐implant diseases (PID), were frequently reported, which subsequently led to implant loss in several cases. These complications not only provide considerable challenges to clinicians but also impose financial burdens on patients, highlighting the importance of prevention and management strategies in view of increasing dental implant procedures (Moraschini et al. 2015). PID manifest in two forms: peri‐implant mucositis and peri‐implantitis. Peri‐implant mucositis is characterized by inflammation limited to the soft tissue around the implant without any associated loss of alveolar bone. This condition is considered reversible, as timely intervention involving the removal of the causative factor can effectively resolve the inflammation. However, without early detection, peri‐implant mucositis may progress into peri‐implantitis, a pathological and irreversible condition around dental implants that manifests as inflammation in the peri‐implant mucosa with progressive loss of surrounding alveolar bone (Berglundh et al. 2018). Due to its increasing incidence and accelerating pattern of progression, peri‐implantitis has emerged as a significant challenge in contemporary implant dentistry. Hence, early diagnosis of peri‐implant mucositis is an effective method to reduce the likelihood of developing peri‐implantitis (Heitz‐Mayfield 2008).

Recently, microRNAs (miRNAs) have emerged as promising molecular biomarkers with the potential to be utilized as diagnostic markers in the field of implant dentistry. miRNAs are a class of small, noncoding RNA molecules that consist of approximately 18–22 nucleotides. They are responsible for coordinating various physiological and pathological processes, including cell proliferation and maturation, apoptosis, regulation of chronic inflammation, and carcinogenesis (Wu et al. 2017). In addition, miRNAs have been identified as significant regulators of bone homeostasis. They exert their effect on the osteoclastogenesis pathway, suggesting a possible role in alveolar bone resorption, a key characteristic of peri‐implantitis (Wu et al. 2017). Additionally, there is supporting evidence indicating that the presence of miRNAs in peri‐implant tissue could serve as an indicator of the ongoing disease processes taking place in the soft tissues surrounding the implant. Consequently, this might become a potentially valuable tool for evaluating the susceptibility of peri‐implant tissues to PID (Menini et al. 2019).

It has been shown that oral fluids, particularly saliva and gingival/peri‐implant crevicular fluid, contain high‐quality miRNAs (Fujimori et al. 2019). Gingival crevicular fluid (GCF) is a transudate derived from serum, present within the gingival sulcus as a result of the host's inflammatory response (Offenbacher et al. 2010). Apse et al. were the first researchers to observe the presence of peri‐implant crevicular fluid (PICF), a comparable fluid found within the peri‐implant crevice (Apse et al. 1989). The biochemical constituents of GCF and PICF undergo alterations corresponding to the inflammatory status, thereby serving as accurate biomarkers for assessing the changes in subjacent tissues (Bhadbhade et al. 2013). Saito and co‐workers first discovered the presence of miRNAs in GCF (Saito et al. 2017). Likewise, Menini et al. reported that it was possible to quantify miRNA expression using PICF (Menini et al. 2021). The collection of GCF/PICF can be carried out efficiently and quickly with minimally invasive procedures compared to gingival tissue samples. Apart from that, information obtained from GCF/PICF is more site‐specific and, therefore, can be directly linked to the diagnosis of each site (Saito et al. 2017).

Research into the role of miRNAs within implant dentistry, while promising, remains sparse, indicating a clear need for additional studies. To date, an animal study using a canine model has been carried out by Wu and co‐workers to identify miRNA expression associated with peri‐implantitis by using gingival soft tissue biopsy. In their study, it was found that let‐7e, miR‐145, and miR‐98 were highly expressed in disease sites compared to healthy, while miR‐146a was under‐expressed. This might reflect the pathological process in peri‐implantitis, given that let‐7e and miR‐145 are related to inflammatory response and bone metabolism, such as osteoclast activity. At the same time, miR‐98 and miR‐146a are involved in the regulation of inflammation‐related signalling pathways (Wu et al. 2017). Having said that, the role of miRNA expressions in PID is not fully understood, but exploring this field might assist researchers in better understanding the pathogenesis of PID. Hence, this research focuses on identifying miR‐98, miR‐145, miR‐146a, and let‐7e in PICF samples from patients exhibiting peri‐implant health, peri‐implant mucositis, and peri‐implantitis and comparing the variations in the expression of these miRNAs among the groups.

## 2 Materials and Methods

### 2.1 Study Design

This cross‐sectional study was designed to assess the expression levels of miR‐98, miR‐145, miR‐146a, and let‐7e biomarkers in PICF samples from patients with peri‐implant health, peri‐implant mucositis, and peri‐implantitis through qPCR analysis. The research was conducted at the Periodontics Specialist Clinic, Faculty of Dentistry, MARA University of Technology, Sungai Buloh Campus, Selangor, Malaysia. Ethical clearance was obtained from the UiTM Research Ethics Committee (REC/08/2021 [MR/659]) and the Medical Research and Ethics Committee (MREC) of the Ministry of Health, Malaysia (NMRR‐21‐1838‐59924 [IIR]), ensuring adherence to the Helsinki Declaration, as revised in 2013. This manuscript was written in accordance with STROBE guidelines for reporting the observational study.

### 2.2 Eligibility Criteria

Individuals with at least one dental implant in function for at least 1 year or have been receiving dental implant treatment/maintenance at the Faculty of Dentistry, UiTM Sungai Buloh and Periodontic Unit, Kota Damansara Dental Clinic, Selangor, were recruited to participate in this study. The study participants included were those diagnosed with peri‐implant health, peri‐implant mucositis, and peri‐implantitis based on specific clinical and radiographic criteria, according to the latest case definition (Berglundh et al. 2018). Exclusion criteria encompassed uncontrolled diabetes mellitus, the use of antimicrobials or anti‐inflammatories within 3 months before the study, smokers, as well as pregnant or lactating mothers.

### 2.3 Sample Size Calculation

A statistical power analysis was performed for sample size estimation using G*Power3.1 software based on data from a published study (Chaparro et al. 2021). The effect size in this study was 0.54, which is considered a large effect according to Cohen's criteria (Cohen 2013). With an *α* = 0.05 and power = 0.80, the projected sample size needed with this effect size is approximately *N* = 39 or 13 samples/group.

### 2.4 Participant's Recruitment

Written consent was clearly informed and signed by all agreed participants. A comprehensive periodontal assessment was conducted by one researcher (N.L.Z.A.) followed by an evaluation of the peri‐implant clinical examination and radiographic evaluation of the individual dental implants. Based on the diagnosis, the dental implants were allocated into three groups, consisting of Group 1 with peri‐implant health, Group 2 with peri‐implant mucositis, and Group 3 with peri‐implantitis. All relevant sociodemographic backgrounds of the participants were recorded in a separate form. Additionally, the baseline demographic and clinical characteristics of each of the dental implants were recorded. Then, the collection of PICF samples was conducted during a subsequent visit.

### 2.5 PICF Collection

The patients were advised to refrain from eating, drinking, or performing vigorous oral hygiene procedures at least 90 min before PICF collection. To avoid contamination of the implant site with saliva, the area was washed with distilled water and isolated with a cotton roll. Then, the site was lightly dried for around 5 s using a three‐way syringe, after which the supragingival plaque was gently removed with a dry cotton pellet, avoiding contact with the gingival margin. After that, PICF was collected at each implant site using an absorption technique described by Saito et al. (2017). In this technique, #30 endodontics paper point (FKG Dentaire SA, Switzerland) was carefully inserted 1–2 mm into the peri‐implant sulcus and left in situ for about 30 s to collect a sufficient volume of PICF without causing trauma to the site. PICF samples that were visibly contaminated with blood were discarded. Several paper points were utilized to collect around 4.8 µL of PICF per implant site. Considering that a single paper point can absorb up to 1.2 µL of PICF, the sample volume was estimated by assessing the absorption levels of the collected strips. Hence, a total of 8–12 paper points were collected from four sites per implant. It is worth noting that each paper point absorbed at least one‐third to half of its capacity, equivalent to 0.4–0.6 µL, before being carefully placed into an Eppendorf tube 400 µL of phosphate buffer saline (PBS). Then, the samples were securely stored at −80°C until further analysis.

### 2.6 RNA Extraction and Isolation

Total RNA was isolated from PICF samples using an miRNeasy Serum/Plasma Advanced Kit (Qiagen, UK), according to the manufacturer's instructions. PICF samples from the control group were pooled together, while PICF samples from Group 2 and Group 3 were individually analyzed. PICF samples were gently thawed on ice and then briefly centrifuged at 5000 g for 10 s at room temperature. For each sample, 200 µL of PICF was transferred into a new 1.5 mL microcentrifuge tube. After that, the samples were mixed with Buffer RPL and Buffer RPP to aid in the process of lysis. Then, 1 µL of spike‐in control, containing UniSp2, UniSp4, and UniSp5 RNA Spike‐in Mix, was added to check for the efficiency of RNA extraction. The clear supernatant was treated with isopropanol and passed through a spin column to bind RNA. Following washing steps using Buffer RWT, Buffer RPE, and 80% ethanol to remove contaminants, RNA was eluted with 20 µL of RNase‐free water. The purified RNA, approximately 18 µL in volume, was then stored at −80°C for subsequent qPCR analysis.

### 2.7 cDNA Synthesis and qPCR

Following optimization of the template RNA, 6 µL of the RNA eluate was reverse‐transcribed into cDNA in 10 µL reactions using the miRCURY LNA Universal Reverse Transcription Kit (catalogue number 339340) from Qiagen, UK. The UniSp6 RNA spike‐in kit was added to check for the efficiency of cDNA synthesis. This cDNA was then diluted in a ratio of 1:30 and used in 10 µL PCR reactions, following the protocol of the miRCURY LNA SYBR Green PCR Kit (Qiagen, UK). The primer assay for specific miRNA targets, namely, miR‐98, miR‐145, miR‐146a, and let‐7e, was designed and ordered from Sigma Aldrich Biotechnology, Brazil. The sequence was adapted from the previous study (Wu et al. 2017). Duplicate real reactions containing 10 µL reaction volumes were performed for the control group, diseased sample (Groups 2 and 3), and no‐template control containing RNase‐free water. Amplification took place in a real‐time qPCR cycler (Applied Biosystems StepOnePlus, Foster City, CA, USA) using 96‐well clear plates. The qPCR involved an initial cycle at 95°C for 2 min, followed by 40 cycles of 95°C for 10 s and 56°C for 60 s. Expression levels of the target miRNAs were normalized against a reference gene (RG), miR‐103a‐3p, and quantification was achieved using the comparative 2^(^^−∆ΔCq)^ method. The amplification plots were analyzed using StepOne Software Version 2.1, which was utilized to determine the quantitative cycle (*C_q_*) values and perform melting curve analysis accurately.

### 2.8 Statistical Analysis

Statistical analysis was conducted using SPSS version 28 (IBM Corp., Armonk, New York, USA). The Shapiro–Wilk test was used to assess the normality of the data distribution. Categorical variables were summarized as frequencies and percentages, while continuous variables were reported as means and standard deviations or medians and interquartile ranges (IQR) based on their distribution. The Kruskal–Wallis test was employed to compare the relative expression of genes across different groups, with the Bonferroni post hoc test used for further comparisons. A *p*‐value of less than 0.05 was considered statistically significant.

## 3 Results

### 3.1 Study Flow Chart

**Figure 1:**
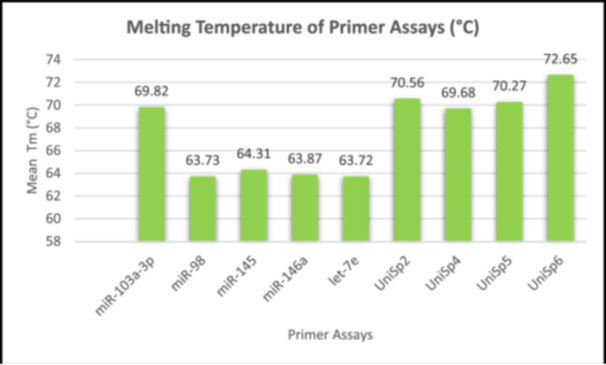
Bar chart of the mean *t_m_* value of the primer assays.

During the enrolment visit from January 2022 until October 2023, 21 participants with 40 dental implants underwent screening through the patient data system. However, two individuals were deemed ineligible and therefore excluded from the study because they were smokers or had been treated with antimicrobial medications. Consequently, the study proceeded with 19 eligible participants who collectively had 38 dental implants. Following that, a comprehensive periodontal assessment was conducted by one researcher (N.L.Z.A.) on the 19 qualified subjects, followed by an evaluation of the peri‐implant clinical examination and radiographic evaluation of the individual dental implants. The clinical examinations that were recorded included modified plaque index (mPI), modified sulcus bleeding index (msBI), pocket probing depth (PPD), clinical attachment level (CAL), and marginal bone level (MBL). Based on the diagnosis, the dental implants were allocated into three groups, consisting of Group 1 with peri‐implant health (*n* = 12), Group 2 with peri‐implant mucositis (*n* = 16), and Group 3 with peri‐implantitis (*n* = 10). The collected PICF samples were stored at −80°C until they were ready for RNA isolation and quantification. For the qPCR analysis, the 12 samples from Group 1 (serving as the control group) were pooled, while the samples from Group 2 and Group 3 were evaluated individually. Finally, a total of 27 samples were subjected to qPCR analysis to detect the presence of specific miRNAs (miR‐98, miR‐145, miR‐146a, and let‐7e) and quantify and compare the level of gene expression among the groups. The study flow chart is shown in Figure 1.

### 3.2 Baseline Demographics and Clinical Characteristics of the Study Participants and the Dental Implants

Table 1 illustrates the descriptive analysis of the baseline sociodemographic information of the study participants. At baseline, the mean age of the participants was 57.6, ranging from 32 to 76 years of age. The majority of participants were of Malay ethnicity, accounting for 78.9% of the total subjects. Additionally, a notable proportion of 21.1% reported the presence of well‐controlled diabetes mellitus (the latest HbA1c value of less than 7%), hypertension, and hypercholesterolemia. Furthermore, a total of six individuals disclosed a previous history of periodontal disease, accounting for 31.6% of the total participants. Table 2 displays the baseline demographic and clinical characteristics of the dental implants. Based on the Fisher Exact test, the types of prosthetic restoration and duration of implant loading demonstrated a significant difference between the groups with *p* = 0.039 and *p* = 0.019, respectively. Table 3 shows the baseline clinical parameters of each group using Kruskal–Wallis test. Based on the results, there was a statistically significant difference observed among the groups in terms of mPI, msBI, PPD, CAL, and MBL measurement with *p* < 0.05.

**Table 1:** Mean *Cq* value of each of the target genes at different concentrations of the template RNA.

Target genes	Concentration of template RNA		
	2 µL	4 µL	6 µL
miR‐103a‐3p (RG)	33.60735	28.64901	26.89181
miR‐98	ND	ND	ND
miR‐145	ND	ND	ND
miR‐146a	ND	ND	ND
Let‐7e	ND	ND	36.17502
UniSp2	21.86884	20.40332	19.51319
UniSP4	29.13471	27.90124	23.36328
UniSp5	36.95537	35.09418	34.2146
UniSp6	23.8098	22.41168	22.30545

**Table 2:** Primer assays for the miRNA of interest.

Target gene	Mature sequence	Assay ID
hsa‐miR‐98	UGAGGUAGUAAGUUGUAUUUGUU	MIRAP00113
hsa‐miR‐145‐5p	GUCCAGUUUUCCCAGGAAUCCCU	MIRAP00180
hsa‐let‐7e‐5p	UGAGGUAGGAGGUUGUAUAGUU	MIRAP00008
hsa‐miR‐146a‐5p	UGAGAACUGAAUUCCAUGGGUU	MIRAP00182

*Note:* Designed and ordered from Sigma Aldrich Biotechnology, Brazil.

**Table 3:** Primer Assay for the Endogenous Control.

Endogenous control	Mature sequence	Assay ID
miR‐103a‐3p	AGCAGCAUUGUACAGGGCUAUGA	YP00204063

*Note:* Designed and ordered from Qiagen, UK.

### 3.3 Efficiency of RNA Extraction and cDNA Synthesis

To assess the effectiveness of RNA extraction and cDNA synthesis, we followed an alternative procedure outlined by Blondal and colleagues that involved incorporating RNA spike‐ins (UniSp2, UniSp4, UniSP5, UniSP6) at the beginning of the RNA isolation process (Blondal et al. 2013). These external RNA controls, not originally present in the samples, serve distinct purposes: UniSp2, UniSp4, and UniSp5 evaluate RNA isolation efficiency, while UniSp6 is specifically used to evaluate cDNA synthesis efficacy. Our results consistently showed successful detection of all RNA spike‐ins, including UniSp2, UniSp4, UniSP5, and UniSP6, in all samples. The average *C_q_* values and standard deviations of the RNA spike‐ins are presented in Table 4.

**Table 4:** Descriptive analysis of baseline demographic of study participants.

Categories	Total (*N* = 19)	
	Mean (range)	*N* (%)
Age	57.57 (32–76)	
Gender		
Male		8 (42.1)
Female		11 (57.9)
Race		
Malay		15 (78.9)
Chinese		3 (15.8)
Indian		1 (5.3)
Medical status		
Healthy		15 (78.9)
Co‐morbidities		4 (21.1)
History of periodontal disease		
No		13 (68.4)
Yes		6 (31.6)
Periodontal diagnosis		
Healthy		1 (5.3)
Gingivitis		6 (31.6)
Gingival inflammation in stable periodontitis		5 (26.3)
Stage III periodontitis		5 (26.3)
Stage IV periodontitis		2 (10.5)
Parafunctional habits		
Yes		0
No		19 (100)

### 3.4 Melting Curve Analysis

**Figure 2:**
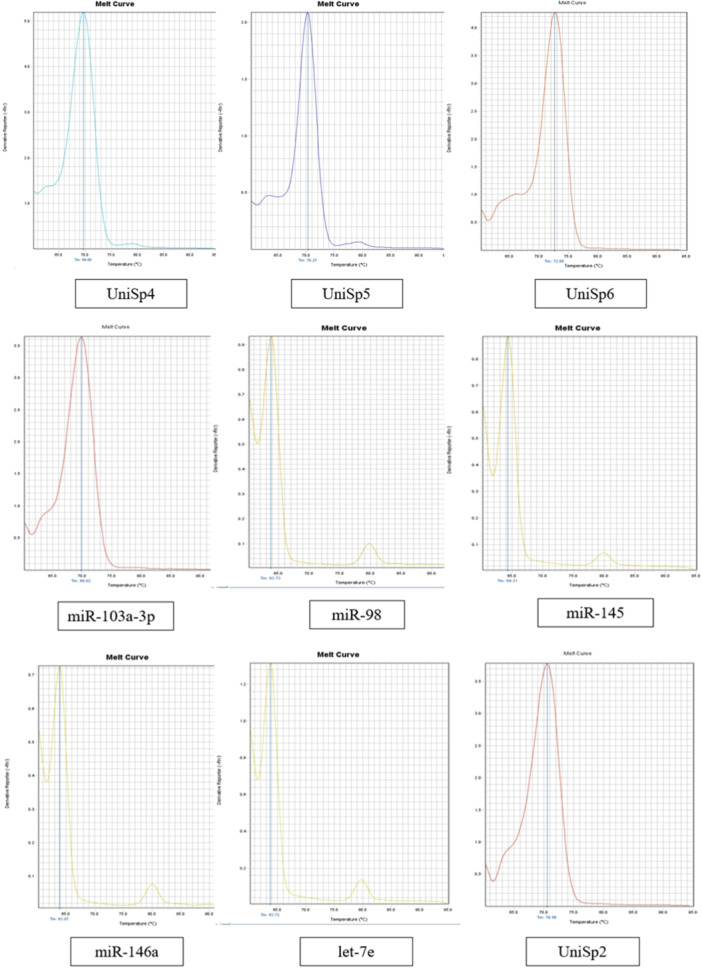
Melting curve analysis of each primer assay.

**Figure 3:**
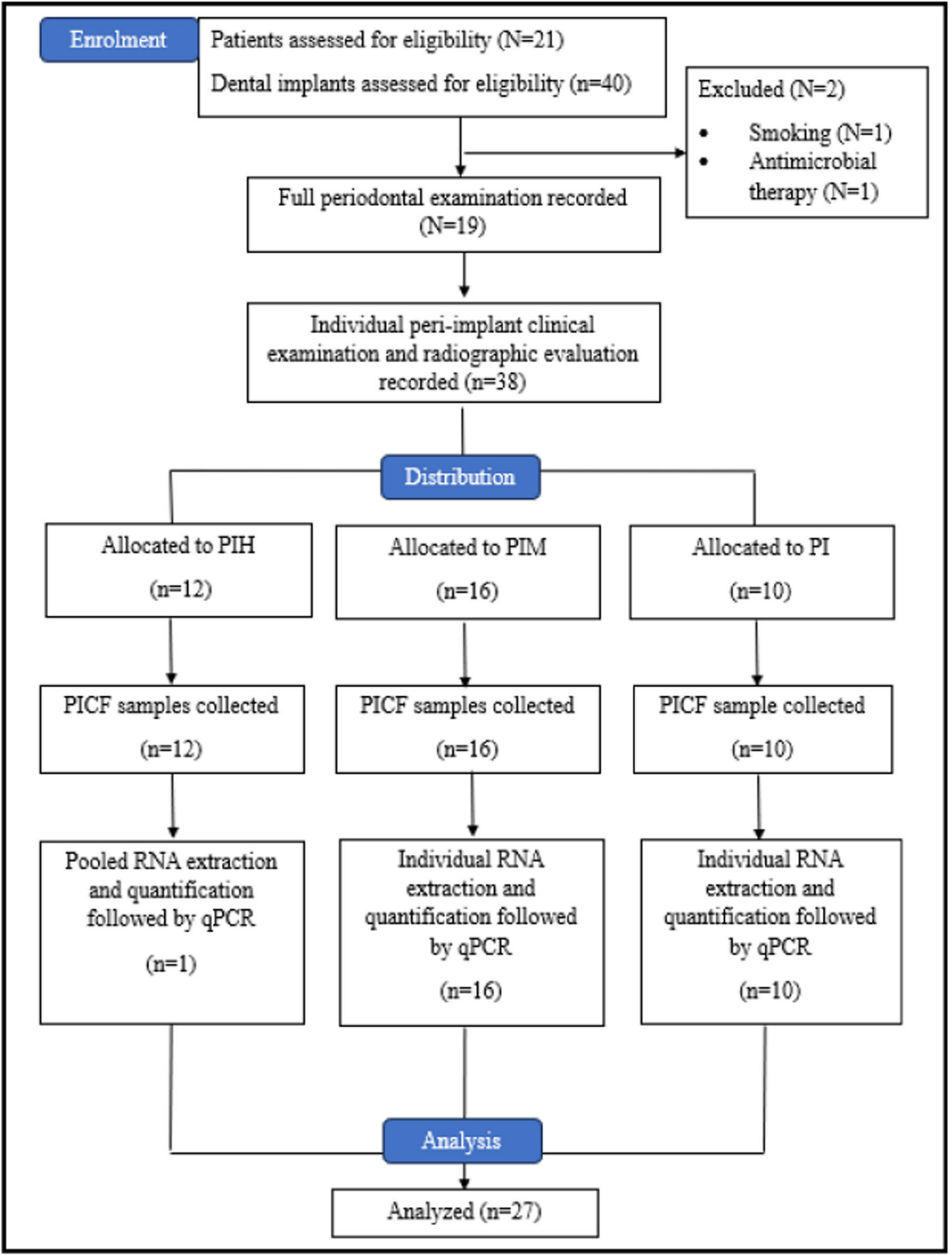
CONSORT flow diagram of the cross‐sectional study.

To determine the specificity of primer assays, melt curve analysis of qPCR analysis was performed. Melting temperature (*T_m_*) is defined as the temperature at which 50% of DNA strands dissociate into single‐stranded DNA (Seo et al. 2022). Based on the analysis illustrated in Figure 2, the RG (miR‐103a‐3p) has a Tm of 69.82°C. This Tm is consistent with the Tm range of the RNA spike‐ins, which fall between 69.68°C and 72.65°C. In contrast, the Tm values for the miRNAs of interest were slightly lower, ranging from 63.72°C to 64.31°C. These Tm values, while lower than that of the RG, were fairly close to each other. Additionally, the melting curve analysis presented in Figure 3 reveals that both the RG and RNA spike‐in assays demonstrated clear, single, sharp peaks indicative of specific amplification. Similarly, the assays for the miRNAs of interest also displayed single sharp peaks. Notably, a small secondary peak is observed in the melt curves for the miRNAs of interest. However, the *T_m_* values of these secondary peaks are considerably different from the primary peaks.

### 3.5 Frequency of Detection of miRNAs of Interest

qPCR analysis was conducted to investigate the quantitative expression pattern of miR‐98, miR‐145, miR‐146a, and let‐7e in PICF samples obtained from individuals with peri‐implant mucositis and peri‐implantitis, with peri‐implant health as a control group. To detect the presence of miRNAs of interest, we employed duplicate samples to calculate the average *C_q_* value of pooled samples from the control group and individual samples from Group 2 and Group 3. A total of 27 samples were subjected to qPCR analysis. Based on the findings of our study, none of the miR‐98, miR‐145, and miR‐146a were identified in any of the samples. Interestingly, only let‐7e biomarkers were consistently detected in the pooled samples of Group 1, as well as in all individual samples from Group 3. However, in Group 2, only 75% (12 out of 16) of the samples exhibited the presence of let‐7e biomarkers. Table 5 and Figure 4 illustrate the frequency of detection of miRNA biomarkers.

**Table 5:** Baseline demographic and clinical characteristics of dental implants.

Variables	PIH (*n* = 12)	PIM (*n* = 16)	PI (*n* = 10)	*p*‐value
		*n* (%)		
Implant location				0.787
Maxillary anterior	4 (33.3)	5 (31.3)	6 (60.0)	
Maxillary posterior	4 (33.3)	7 (43.8)	2 (20.0)	
Mandibular posterior	4 (33.3)	4 (25.0)	2 (20.0)	
Type of prosthetic restoration				0.039*
Single	11 (91.7)	10 (62.5)	4 (40.0)	
Connected crown	0 (0.0)	0 (0.0)	2 (20.0)	
Implant‐supported bridges	1 (8.3)	5 (37.5)	4 (40.0)	
Type of prosthetic retention				0.054
Screw‐retained	6 (50.0)	9 (56.3)	1 (10.0)	
Cement‐retained	6 (50.0)	7 (43.7)	9 (90.0)	
Type of opposing dentition				0.342
Natural teeth	11 (91.7)	11 (68.8)	10 (100.0)	
Crown	1 (8.3)	3 (18.8)	0 (0.0)	
No opposing teeth	0 (0.0)	2 (12.4)	0 (0.0)	
Duration of implant loading				0.019*
1–5 years	8 (66.7)	9 (56.3)	1 (10.0)	
More than 5 years	4 (33.3)	7 (43.7)	9 (90.0)	

*Significant at the level of 0.05. The non‐normality assumption is fulfilled. Fisher's Exact Test was applied.

**Figure ‎4:**
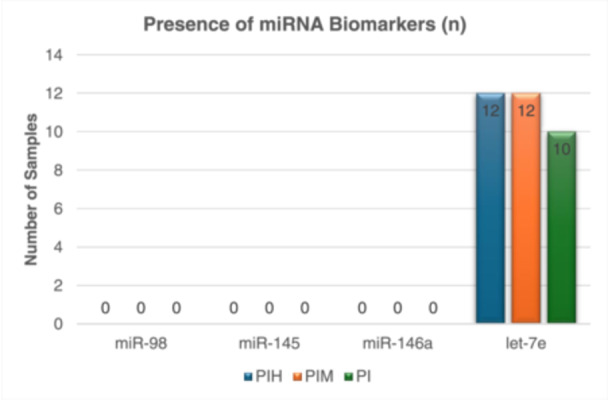
Bar chart of the presence of miRNA biomarkers in PIH, PIM, and PI groups.

### 3.6 Fold Changes in let‐7e Expression

To assess the changes in gene expression levels, fold change analysis was employed, which is commonly used in qPCR experiments. The RG, miR‐103a‐3p, was utilized as a normalization control, and the mean *C_q_* value was determined for each sample. To quantify the miRNA expression, Δ*C_q_* was calculated using this formula:

ΔCq=mean Cqof let−7e–mean Cqof RG.

To assess the variations of let‐7e expression among three different groups, the ΔΔ*C_q_* method was employed. This method involves calculating the difference in Δ*C_q_* values between the diseased sample and the Δ*C_q_* values of the control. Subsequently, the relative expression of let‐7e was then determined using the following formula:

Fold changes=2^−ΔΔCq.

**Figure 5:**
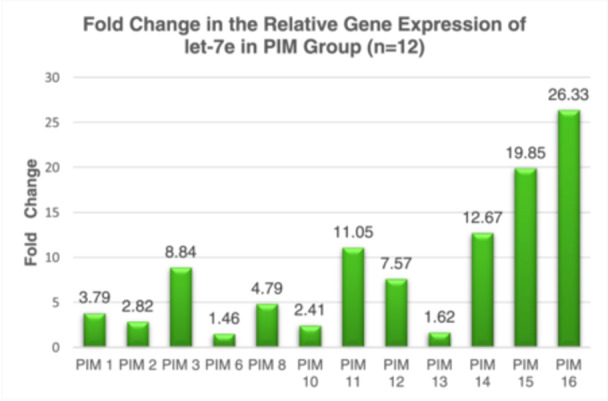
Bar chart of fold change in the relative gene expression of let‐7e among individual samples in the PIM group.

Table 6 and Figure 5 illustrate the fold changes in let‐7e gene expression across the 3 groups. Based on the findings, let‐7e was upregulated approximately eightfold in Group 2 and 94‐fold in Group 3. Subsequently, a Kruskal–Wallis test was conducted to assess differences in let‐7e expression levels among the groups (Table 7). The findings revealed a statistically significant difference in let‐7e expression across the groups, with a Kruskal–Wallis *H* statistic of 25.825 and a *p*‐value of less than 0.001. A post‐hoc analysis with a Bonferroni‐adjusted alpha level of 0.05 was employed for pairwise group comparisons. Significant differences were observed both between Group 1 and Group 3, and Group 2 and Group 3, each with *p*‐values less than 0.05 (Tables 8 and 9).

**Table 6:** Baseline clinical parameters using Kruskal–Wallis test.

Variables	Median (IQR)			H (*df*)	*p*‐value
	PIH	PIM	PI		
mPI	0.38 (0.69)	0.50 (0.69)	1.25 (0.63)	10.61 (2)	0.005*
msBI	0.00 (0.44)	0.50 (0.69)	1.75 (1.25)	27.18 (2)	< 0.001*
PPD	2.83 (0.57)	3.05 (0.75)	4.17 (1.25)	19.56 (2)	< 0.001*
CAL	3.08 (0.76)	3.42(1.25)	4.50 (1.75)	16.01 (2)	< 0.001*
MBL	0.45 (0.68)	0.48 (1.43)	3.83 (1.58)	21.64 (2)	< 0.001*

*Note:* The non‐normality assumption is fulfilled. Kruskal–Wallis test was applied.

* Significant at the level of 0.05.

**Table 7:** Frequency of detection of miRNA biomarkers.

Groups (*N*)	Frequency of detection of miRNA biomarkers, *n* (%)			
	miR‐98	miR‐145	miR‐146a	let‐7e
PIH (12)	0 (0)	0 (0)	0 (0)	12 (100)
PIM (16)	0 (0)	0 (0)	0 (0)	12 (75)
PI (10)	0 (0)	0 (0)	0 (0)	10 (100)

**Table 8:** Comparison of let‐7e gene expression among groups.

Variables	Median (IQR)			H (*df*)	*p*‐value
	PIH	PIM	PI		
Let‐7e expression	1.00 (0.00)	2.41 (8.8)	60.62 (88.7)	25.83 (2)	< 0.001*

*Note:* The non‐normality assumption is fulfilled. Kruskal–Wallis test was applied.

* Significant at the level of 0.05.

**Table 9:** Pairwise comparison of the significant results using bonferroni correction.

Variables	Pairwise comparison	*p*‐value
Let‐7e expression	PIH versus PI, PIM versus PI	< 0.05

*Note:* While the comparison of the other pairs, *p* > 0.05.

## 4 Discussion

**Figure 6:**
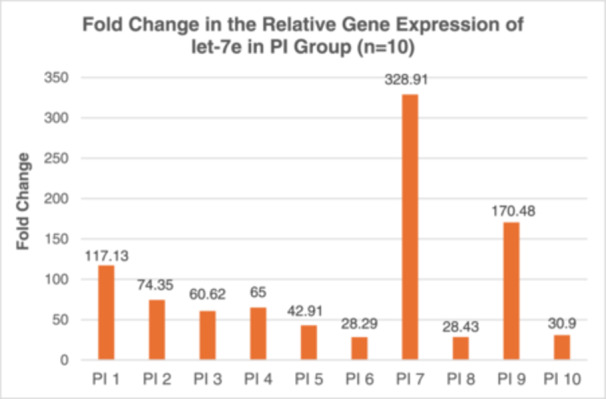
Bar chart of fold change in the relative gene expression of let‐7e among individual samples in the PI group.

**Figure 7:**
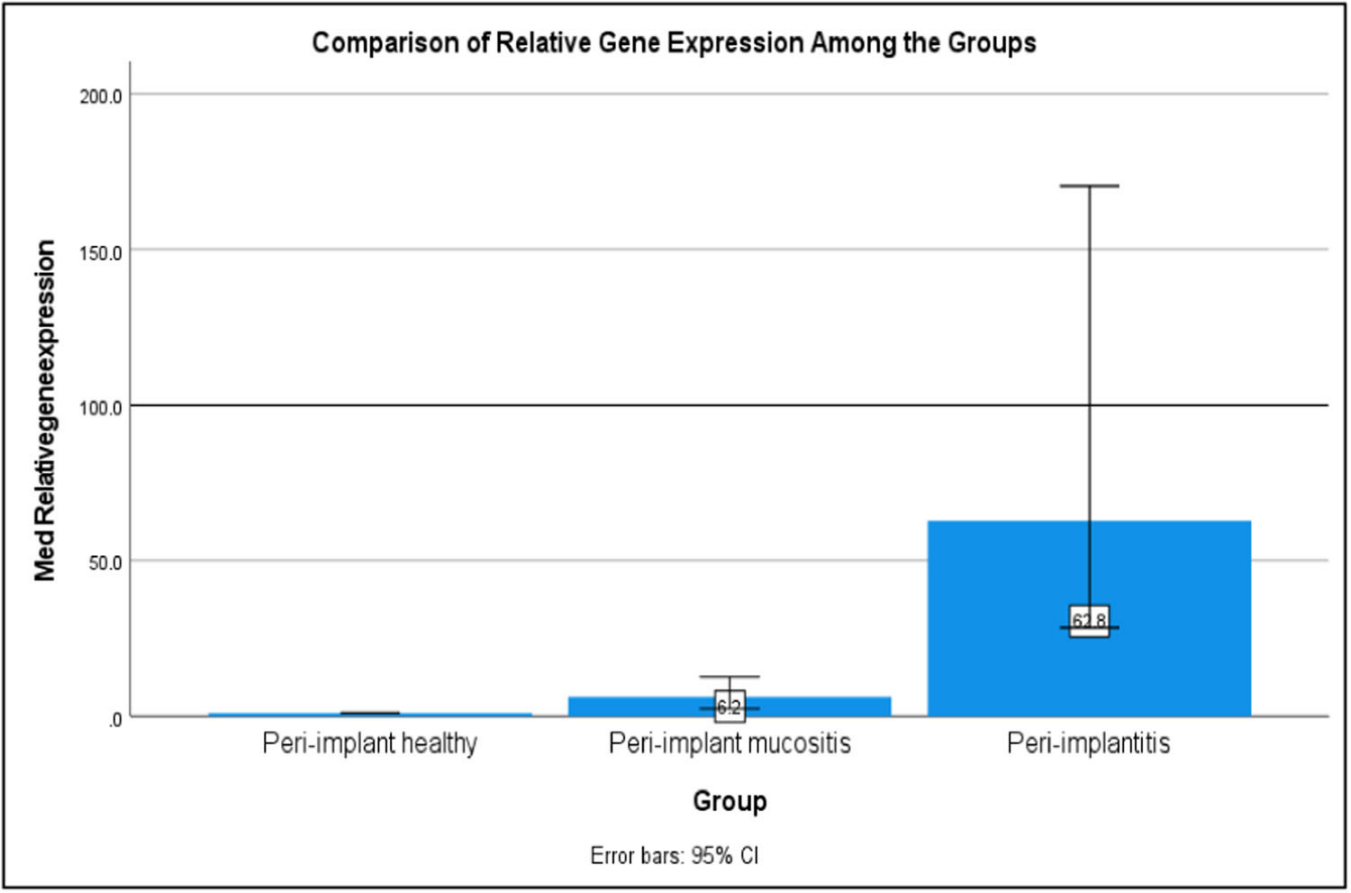
Comparison of the median relative gene expression of let‐7e among groups.

**Figure 8:**
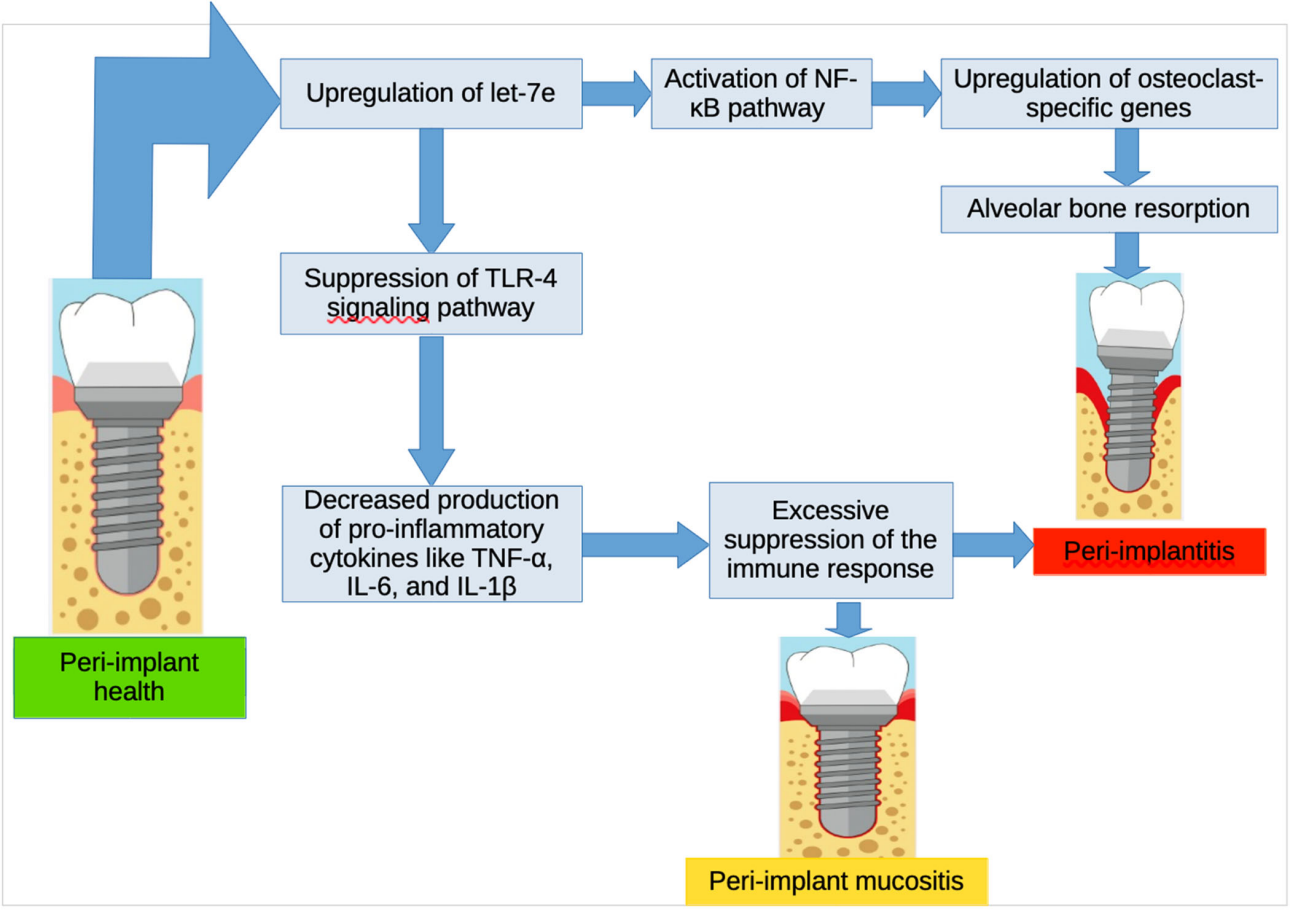
Possible mechanism of let‐7e in the development of peri‐implant diseases.

In this investigation, we analyzed the expression patterns of specific miRNAs, including miR‐98, miR‐145, miR‐146a, and let‐7e, in relation to PID. Our results revealed that miR‐98, miR‐145, and miR‐146a were absent in all tested samples. (Figures 6, 7, 8). Notably, only the let‐7e biomarkers were found consistently in both the control group's pooled samples and all samples from Group 3. However, let‐7e was detected in 75% of Group 2 samples. This contrasts with studies by Wu and co‐workers who identified these miRNAs in canine gingival tissues (Wu et al. 2017) and Menini et al. who found them in human PICF samples (Menini et al. 2021). The discrepancies in miRNA detection between this study and others might be due to several factors. One primary difference is the type of samples used. Wu et al. used gingival tissues, which might naturally have higher miRNA levels due to their tissue origin (Wu et al. 2017), whereas our study used PICF, a less invasive but potentially less concentrated miRNA source compared to those derived from tissue biopsies. Additionally, the choice of analytical techniques could influence outcomes. We employed qPCR to analyze miRNAs, focusing on specific targets, whereas Menini et al. used a microarray technique, which screens for a broad array of miRNAs and could detect even low‐concentration miRNAs across numerous samples (Liu et al. 2004). The choice of qPCR in our study, focusing on specific miRNAs based on prior hypotheses, may have limited our ability to detect the broad range of miRNAs present, especially those at lower concentrations. Another factor to consider is the possibility of suboptimal primer design impacting the efficiency of miRNA amplification, leading to inefficient or nonspecific amplification. The specificity and efficiency of primer‐miRNA binding are critical variables that can influence the qPCR outcomes. These factors are essential because they determine how well the primers can anneal to the target miRNA sequences, directly influencing the amplification process's accuracy and specificity. Zhao et al. emphasized that when using the relative quantification method for data analysis, optimizing qPCR conditions for each reference and target gene should be performed (Zhao et al. 2021). Hence, we conducted melt curve analysis to ensure primer specificity and to detect any primer‐dimer formations or nonspecific products. A sharp, single peak in melt curve analysis usually indicates specific amplification, while multiple peaks suggest nonspecific reactions (Seo et al. 2022). This technique serves as an essential quality control measure, enabling the detection of nonspecific binding or amplification that could compromise the accuracy of qPCR results. For future research, enhancing primer validation processes, such as testing primer efficiency under various conditions, could be crucial, although financial constraints limited this in our study. Additionally, our inability to detect certain miRNAs might reflect limitations in assay sensitivity or differences in disease pathology among our samples, suggesting that miRNA profiles may vary significantly between populations and disease states. This complexity highlighting the need for more detailed investigations into miRNA regulation in different disease contexts.

Interestingly, our study stands out by focusing on let‐7e. To our knowledge, this is the first human study to explore let‐7e gene expression in the context of PID. The prior research by Wu et al which identified let‐7e in gingival tissues of a canine model, serves as the only animal study highlighting its potential relevance (Wu et al. 2017). The consistent detection of let‐7e in cases of peri‐implantitis and its significant presence in most peri‐implant mucositis samples in our study suggests a potential role for let‐7e in the inflammatory processes associated with PID. This finding corroborates the theory proposed by Wu et al. concerning the role of let‐7e in the inflammatory process and disease progression (Wu et al. 2017). In addition, it might serve as a more sensitive marker for identifying the presence and progression of PID than other miRNAs we studied. However, this may need to be validated with a large‐scale prospective cohort study.

In our study, the absence of let‐7e biomarkers in 25% of the peri‐implant mucositis samples warrants consideration of several potential influencing factors, especially in terms of sample collection and processing, which are vital for preserving miRNA stability. To address variability, we standardized sample collection times between 9 a.m. and 11 a.m. for all participants, aiming to minimize any biases and ensure consistency across samples. Additionally, we adopted a standardized method for collecting PICF using endodontic paper points, as previously outlined by Saito and co‐workers (Saito et al. 2017) to reduce discrepancies in sample acquisition. To reduce the risk of RNA degradation during transportation, PICF samples were immediately placed in an ice‐filled box and stored at −80°C until analysis. This protocol was designed to enhance RNA stability and maintain the integrity of our samples for accurate evaluation. Another critical aspect to consider is the quality of the PICF samples themselves. Factors such as RNA degradation or the presence of inhibitory substances could severely impede miRNA detection. However, the consistent detection of RNA spike‐ins during the qPCR assays confirms the effectiveness of our RNA isolation and cDNA synthesis procedures. Furthermore, the biological variability among participants and differences in disease states might also influence miRNA expression levels, adding complexity to interpreting our findings. Additionally, the sensitivity of the qPCR techniques used, particularly for low‐abundance miRNAs, must be acknowledged as a potential limitation affecting the detection of let‐7e biomarkers in some samples.

Our research identified a marked increase in let‐7e expression, revealing an eightfold increase in peri‐implant mucositis and a striking 94‐fold rise in peri‐implantitis compared to controls. These results indicate a significant correlation between let‐7e levels and the severity of PID, suggesting that let‐7e could be crucial in the inflammatory dynamics of these disorders. This aligns with Wu et al.'s findings, which noted a 1.5‐fold increase in let‐7e in peri‐implantitis cases within animal models (Wu et al. 2017). Additionally, research by Hwa Lee et al. reported over a fourfold increase in let‐7e expression in periodontitis patients, further associating it with periodontal inflammation (Lee et al. 2011). Our observations of notably higher let‐7e expression—far surpassing those reported in prior studies—could be due to differences in sample characteristics and the stages of disease, whether they are active or in remission. This is evident from the wide range of fold changes observed in peri‐implantitis, ranging from 27‐fold to as high as 300‐fold. Interestingly, our study explores new ground by examining let‐7e expression in peri‐implant mucositis, addressing a significant gap in existing literature. We found that let‐7e is also significantly upregulated in the peri‐implant mucositis group, supporting the theory that let‐7e is involved in the inflammatory response crucial to PID. Additionally, the pronounced increase in let‐7e expression in peri‐implantitis may underscore its role in more severe stages of the disease, characterized by increased inflammation and bone loss.

When looking at the potential mechanism through which let‐7e might influence PID development, research have shown that let‐7e is a key regulator of endothelial function and inflammation (Lin et al. 2017). Guan et al. found that let‐7e controls endotoxin sensitivity and tolerance of macrophages by targeting TLR‐4. This suggests that let‐7e plays a role in regulating the immune response and inflammation. The same study also identified that overexpression of let‐7e induces activation of Th1 and Th17 cells, exacerbating experimental autoimmune encephalomyelitis by targeting IL‐10 (Guan et al. 2013). In addition, let‐7e may play a pivotal role in bone metabolism, particularly through its influence on the inflammatory response and osteoclastogenesis. They can amplify inflammatory signals in endothelial cells and promote the differentiation of monocytes into osteoclasts, a key process in bone resorption and remodeling. It does so by facilitating the translocation of the NF‐κB transcription factor into the nucleus, thereby activating the NF‐κB pathway. The activation of NF‐κB leads to the upregulation of osteoclast‐specific genes and the downregulation of genes that are inappropriate for osteoclast function (de la Rica et al. 2015). Given that bone resorption is a hallmark of peri‐implantitis, understanding the molecular mechanisms by which let‐7e regulates osteoclastogenesis and inflammation may provide valuable insights into the development and progression of the disease. Nevertheless, there is a scarcity of conclusive information indicating the precise function of let‐7e in developing PID. Although the above‐cited studies have indicated the role of let‐7e in controlling inflammation, immunological responses, and bone metabolism, its specific connection with PID has yet to be fully understood. The comprehensive understanding of how let‐7e contributes to the pathogenesis of PID remains a subject of ongoing research, with the potential to uncover novel therapeutic targets to mitigate these conditions and subsequently lead to long‐term preservation of dental implant health.

Although the results are promising, there are several limitations of our study. One of the main challenges we faced was the restricted number of samples available for analysis. Despite efforts to recruit participants from two different centers, we fell short of our initial target of 39 dental implants, aiming for 13 samples per group. While we reached our target number for the peri‐implant mucositis group, finding enough samples for the peri‐implant healthy and peri‐implantitis groups proved difficult, as these conditions are less common. Additionally, there was a noticeable imbalance in the ethnic distribution of our participants, with 78.9% being of Malay ethnicity. This limitation in sample size and imbalanced ethnic distribution potentially constrains the statistical power of our findings and may limit the generalizability of the results. Moreover, due to financial limitations, our study faced constraints that led to the omission of crucial steps, notably the optimization of primers and the selection of multiple RGs. Primer optimization is vital for ensuring the specificity and efficiency of miRNA amplification, directly influencing the reliability of qPCR results. Similarly, the use of multiple RGs is recommended for accurate normalization of qPCR data, enhancing the validity of the findings.

## 5 Conclusion

Within the limitations of our study, we conclude that miRNAs, particularly let‐7e, may play a significant role in the development and progression of PID, highlighting the need for further in‐depth investigation. Additionally, the successful detection of let‐7e in PICF established a foundation for utilizing PICF as a medium for noninvasive, site‐specific liquid biopsies, particularly in implant dentistry. This noninvasive diagnostic method has the potential to be adopted in clinical settings, offering a personalized diagnostic that aligns with the principles of personalized medicine. This approach not only aligns with the growing trend towards minimally invasive diagnostic procedures but also highlights the importance of early detection and intervention in enhancing outcomes for patients with PID.

## Author Contributions

M.F.H.H., N.L.Z.A. and F.H.A.B. conceived and designed the project. N.L.Z.A. and. N.L. acquired the data. N.L.Z.A. M.F.H.H. and F.H.A.B. analyzed and interpreted the data. N.L.Z.A. and M.F.H.H. wrote the paper.

## Conflicts of Interest

The authors declare no conflicts of interest.

## Data Availability

The data that support the findings of this study are available from the corresponding author upon reasonable request.
